# Obituary

**Published:** 1877-05

**Authors:** 


### Obituary.

### PROF. JOSEPH W. FREER.

Joseph W. Freer was born at Fort Ann, Washington Co., N. Y., Aug. 19, 1816, from Holland blood on the father's side, and from the English Puritans of New England on the mother's. He worked on his father's farm until his nineteenth year, acquiring during this time but the rudiments of learning. He then entered a store in a neighboring village, in the capacity of clerk. He remained here but a short time, and in June, 1836, he migrated to Illinois. Here he devoted himself to agriculture, making a claim on the Calumet River, not far from Chicago, but he was obliged to abandon it in consequence of sickness. He then opened a farm in Will County, Ill., in which he was among the earliest settlers, where he remained until 1846. He was married in 1844. His wife died in 1845, leaving to him an infant son. He was dissatisfied with the medical treatment of his wife in her last sickness, and this circumstance made him determine to study medicine. With limited literary attainments, and with his hard preliminary experience in life, when thirty years of age, he made his way to Chicago, and placed himself as a private pupil in the office of Daniel Brainard. He was graduated from Rush Medical College in the class of 1848–49. He again married in 1849, and leaves at his death his widow and four surviving children. Soon after his graduation, he was appointed demonstrator of anatomy in Rush Medical College, and when Professor Herrick retired from the chair of anatomy in 1855, Dr. Freer was appointed to his place. In 1859 he was transferred to the chair of physiology and military surgery. When Dr. Blaney resigned the presidency of the school during the winter of 1869 and 1870, Dr. Freer was elected to the position, which he held to the time of his death. At the close of the recent session of lectures in the college he seemed enfeebled by the hard work of the winter. Soon after, he stood several hours in the dissecting-room of the college, when it was imperfectly heated, giving his son instructions in dissection, and from this exposure he took a severe cold, which sent him to a sick bed on the 1st of March, from which he passed to his final rest on the 12th of April following.

His symptoms indicated meningeal inflammation at the base of the brain, and the autopsy proved the accuracy of the diagnosis. Prof. Freer was a self-reliant man, of marked individuality of character. He held all sham and pretense, and falsehood in the garb of truth, in hearty detestation. His opinions of men and events were open to the world, and what he believed he feared not to utter. He made no great claims for himself, but was content to let his life speak for him. His mental qualities were solid and strong, but not brilliant. He had a clear judgment, and great perseverance, and viewing an object before him worthy to attain, he pursued it with a tireless purpose. He led a pure, temperate life of integrity, and was moved by worthy motives. He was sensitively honorable in his relations to the profession. He was honest with his patients, and gave to the consideration of their cases careful study and assiduous attention, and both in medicine and surgery was eminently conservative in his treatment, a practice which added largely to his usefulness.

Though he was always a laborious practitioner, he loved the science of his profession better than its practice, and this doubtless contributed much to his attainments and success. As a teacher, he mastered his subject, and presented it in clear, simple and forcible language, and never submerged his thoughts in superfluous words. He stood among the representative men of his profession in America, and his example is of great value to the young, and is abundantly worthy of their imitation, while to those who have long wrought well in the profession, it lends reflected honor. Such men add dignity to cities and States in which they labor, and any community that holds them in its midst may well give thanks.

